# Infertility and risk of breast cancer in men: a national case–control study in England and Wales

**DOI:** 10.1186/s13058-022-01517-z

**Published:** 2022-05-17

**Authors:** Anthony J. Swerdlow, Cydney Bruce, Rosie Cooke, Penny Coulson, Michael E. Jones

**Affiliations:** 1 Division of Genetics and Epidemiology, The Institute of Cancer Research, 15 Cotswold Road, Sutton, SM2 5NG UK; 2 Division of Breast Cancer Research, The Institute of Cancer Research, London, UK; 3 Nottingham Clinical Trials Unit, School of Medicine, University of Nottingham, Nottingham, UK; 4 Department of Oncology, University of Oxford, Oxford, UK

**Keywords:** Infertility, Breast cancer, Male, Case–control

## Abstract

**Purpose:**

Breast cancer is uncommon in men and its aetiology is largely unknown, reflecting the limited size of studies thus far conducted. In general, number of children fathered has been found a risk factor inconsistently, and infertility not. We therefore investigated in a case–control study, the relation of risk of breast cancer in men to infertility and number of children.

**Patients and methods:**

We conducted a national case–control study in England and Wales, interviewing 1998 cases incident 2005–17 and 1597 male controls, which included questions on infertility and offspring.

**Results:**

Risk of breast cancer was statistically significantly associated with male-origin infertility (OR = 2.03 (95% confidence interval (CI) 1.18–3.49)) but not if a couple’s infertility had been diagnosed as of origin from the female partner (OR = 0.86 (0.51–1.45)). Risk was statistically significantly raised for men who had not fathered any children (OR = 1.50 (95% CI 1.21–1.86)) compared with men who were fathers. These associations were statistically significantly present for invasive tumours but not statistically significant for in situ tumours.

**Conclusion:**

Our data give strong evidence that risk of breast cancer is increased for men who are infertile. The reason is not clear and needs investigation.

## Introduction

Breast cancer is comparatively rare in men, and its aetiology is largely unknown. There are several commonalities with the disease in women, including a genetic component with several specific genes and SNPs known [1] and a relation to anthropometric factors [2]. There is a very high risk in men with Klinefelter syndrome [3], and this, along with the relation to anthropometrics, suggests that sex hormone-related factors might be involved, as they are in women, and a raised risk of breast cancer has been found in the only cohort analysis able to examine prior oestradiol levels in men who subsequently developed breast cancer [4]. In women, reproductive-related factors are important in breast cancer aetiology [5], notably a reduced risk in parous women, and although that relates to the hormonal consequences of parity, which could not plausibly apply in men, it nevertheless seems worth investigating whether male fertility relates to male breast cancer risk. Klinefelter syndrome is associated with infertility [6], and there is some, but not definitive, evidence that testicular abnormalities may also be associated with male breast cancer risk [2]. Infertility is generally defined as the inability to conceive after at least 12 months of regular unprotected sexual intercourse and can be of male origin or female or contributions from both [7].

Investigation of the relation of male infertility and reproductive history to breast cancer risk has been hampered, however, by the rarity of the tumour. Few studies have investigated infertility [8–10] or reproductive history [2, 11–15], the largest based on 227 cases of whom 7 reported male infertility [8]. A pooled analysis has been published [2], but is difficult to interpret because the small studies aggregated had very varied case and control selection criteria, and varied also in definitions of infertility. No significant relation to infertility was found, but there was a significant relation to whether the subject had any children. We have conducted a case–control study in England and Wales covering almost 2000 cases nationally incident over a 12.7 year period and present here the results relating to fertility and children.

## Materials and methods

The study is of case–control design, with potential cases being all male residents of England and Wales with in situ or invasive breast cancer diagnosed in these countries at ages < 80 years during 1 January 2005 to 31 August 2017. These cases were identified from clinician reports to us and comprehensive listings from national population-based cancer registries.

Since attempts to recruit population-based controls now give very low and selective recruitment rates [16, 17], we recruited controls nationally from two sources for which compliance was much higher and made comparisons between the two sources to assess potential bias. The first source was male non-blood relatives of the cases, ascertained by asking the cases about such relatives and then selecting one or more on the basis of stratum-matching on age and geographic region. Secondly, we recruited as controls the husbands of women participating in the Generations cohort study [18], again stratum-matched to cases, and approached via their wives. Since for all of the analyses in the current paper, the two control groups gave results in the same direction, we present in this paper the results for the two groups of controls combined.

Potential cases and controls were asked if they would take part in the study, and if so were interviewed, usually in their homes, by trained research nurse interviewers using a structured questionnaire. The nurses also took a blood sample (or if not possible, a saliva sample) for genetic analyses. The questionnaire enquired about demographic factors and potential risk factors for breast cancer including infertility and offspring. For infertility, we asked whether the subject or their partner had “ever had problems trying to have children for which you or she went to a doctor or infertility clinic’’ and if so we asked for the outcome of the consultation, with responses recorded according to which partner(s) were stated to be the cause of the infertility, and if the case was stated to be infertile, what treatment if any they had received. For offspring, we asked about each biological child the man had fathered, including any who had died.

Analyses of the study data were by standard methods for case–control studies [19], calculating odds ratios (as estimates of relative risks) adjusted for ‘index’ age, year of interview, socio-economic status (residential-based ‘Acorn’ score) [20], marital status and region of residence. The index age for cases was the age at diagnosis, and for controls was an equivalent age derived by calculating for each calendar year of interview, the mean duration for cases from cancer diagnosis to interview, and then subtracting this duration from the age at interview of each control interviewed in that calendar year. Linear trends in risk by exposure level were analysed as continuous variables [19]. We took *p* < 0.05 in a two-sided test to indicate statistical significance.

To examine misclassification and potential confounding, we conducted several sensitivity analyses: excluding subjects whose quality of responses to the overall questionnaires were rated by the interviewer as ‘not well’ or ‘very poorly’; excluding subjects with Klinefelter syndrome, and then excluding also those with other factors that are potential confounders, namely certain testicular conditions, severe obesity, and previous primary cancers that might have involved chest radiotherapy and that directly or via their treatments might have affected fertility; and analyses restricted to married men, as an alternative to adjustment for marital status, since one of the two control groups (the ‘Generations Study’ controls) was by consequence of its source married. We also conducted sensitivity analyses adjusting additionally for alcohol consumption, smoking, family history of breast cancer, and liver disease, in case these might be confounders, although there is no strong evidence that they are.

## Results

From the cancer registries and consultant notifications, we identified 3187 men resident in England and Wales diagnosed during the study period with breast cancer at ages under 80 years. Of these, 433 died (427) or emigrated (6) before we could make contact with them, for 21 we could not identify the consultant or the consultant did not participate, for 28 the consultant deemed the patient unsuitable to be approached, and 707 did not reply to our invitation or declined to participate. The remaining 1998 (62.7%) were interviewed. They were mainly (Table 1) aged 60 years or older at diagnosis, and the tumours were mostly invasive (92.0%) rather than in situ (8.0%), and largely oestrogen receptor positive (98.5% of those with known status). Cases tended to be older than controls, of higher socio-economic status, less often married, less often from the south of England, and interviewed more recently (Table 1): all of these relations except region were statistically significant, and these factors were adjusted for in the risk analyses.

**Table 1:** Descriptive characteristics of male breast cancer cases and controls participating in the study

Characteristic	Cases		Generations study husband controls		Non-blood relative controls		All controls	
	No.	%	No.	%	No.	%	No.	%
Index age (years)^a^								
< 40	47	(2.4)	8	(0.8)	76	(12.4)	84	(5.3)
40–9	159	(8.0)	13	(1.3)	135	(22.1)	148	(9.3)
50–9	385	(19.2)	118	(12.0)	165	(26.9)	283	(17.7)
60–9	729	(36.5)	564	(57.3)	162	(26.4)	726	(45.5)
70–9	678	(33.9)	281	(28.6)	75	(12.2)	356	(22.3)
Socio-economic group (ACORN)^b^								
1 (highest)	710	(35.5)	594	(60.4)	271	(44.2)	865	(54.2)
2	120	(6.0)	35	(3.5)	25	(4.1)	60	(3.8)
3	633	(32.2)	279	(28.4)	204	(33.3)	483	(30.2)
4	318	(15.9)	57	(5.8)	77	(12.5)	134	(8.4)
5 (lowest)	183	(9.2)	12	(1.2)	32	(5.2)	44	(2.7)
Uncategorised^c^	24	(1.2)	7	(0.7)	4	(0.7)	11	(0.7)
Region of residence								
North	375	(18.8)	172	(17.5)	91	(14.5)	263	(16.5)
North-West	368	(18.4)	193	(19.6)	98	(15.6)	291	(18.2)
Mids, East and Wales	379	(18.9)	154	(15.7)	131	(20.9)	285	(17.8)
London & SE	465	(23.3)	270	(27.4)	159	(25.3)	429	(26.9)
South West	411	(20.6)	195	(19.8)	149	(23.7)	344	(21.6)
Year of interview								
2007–9	447	(22.4)	214	(21.7)	229	(37.4)	443	(27.7)
2010–14	807	(40.4)	533	(54.2)	245	(40.0)	778	(48.7)
2015–20	744	(37.2)	237	(24.1)	139	(22.7)	376	(23.6)
Marital status								
Married or cohabiting	1600	(80.1)	984	(100.0)	556	(90.7)	1540	(96.4)
Not married	398	(19.9)	0	(0.0)	57	(9.3)	57	(3.6)
Year of diagnosis								
2005–9	682	(34.1)						
2010–14	851	(42.6)						
2015–17	465	(23.3)						
Breast cancer								
Invasive	1838	(92.0)						
In situ	160	(8.0)						
ER								
+ ve	1844	(92.3)						
− ve	28	(1.4)						
Not known	126	(6.3)						
Total	1998	(100)	984	(100)	613	(100)	1597	(100)

ER, Oestrogen receptor

^a^Age at diagnosis of cases; equivalent age for controls (see Methods)

^b^Acorn score based on postcode of residence [20]

^c^Geographic areas not covered by Acorn (Isle of Man, Channel Islands), and residence in an institution or other non-household location

We approached 828 men to be non-blood relative controls, of whom 613 (74.0%) participated, and we approached 1,109 potential Generations Study controls, of whom 984 (88.7%) participated.

One hundred and twelve (5.6%) cases and 80 (5.0%) controls reported that they had had infertility problems for which they or their partner had consulted a doctor or infertility clinic (Table 2). The prevalence of infertility did not vary by age (controls *P* = 0.90; cases *P* = 0.39). The odds ratio for ever-infertility was statistically non-significantly raised (OR = 1.29, 95% confidence interval (CI) 0.94–1.77). When analysed by outcome of the infertility consultation, there was a statistically significantly raised risk for men who said that they were diagnosed as the source of the couple’s infertility (OR = 2.03 (1.18–3.49)), but not for those whose partner was the source of the infertility (OR = 0.86 (0.51–1.45)) or for whom no source was identified (OR = 1.26 (0.71–2.24)). The same pattern was true in analyses excluding men with Klinefelter syndrome (infertility OR = 1.23 (0.89–1.69); self as source of infertility (OR = 1.81 (1.02–3.20)) and in analyses also excluding men with potentially confounding conditions (Table 2). Analyses restricted to married men were based on somewhat smaller numbers (1600 cases, 1539 controls), but showed similar results, with a statistically significant risk (*p* = 0.02) for men who were themselves infertile (not in Table). Risk was also statistically significantly raised if we aggregated men who were either diagnosed as the source or said the source was unknown but they had been diagnosed with a low sperm count (OR = 2.17 (1.29–3.67), *P* = 0.004, not in table).

**Table 2:** Risk of breast cancer in men in relation to infertility

Infertility	Cases No. %		Controls No. %		Odds ratio^a^ (95% CI) All subjects	*P*	Odds ratio^a^ (95% CI) excluding men with potentially confounding conditions^b^	*P*
No	1810	90.6	1503	94.1	1.00		1.00	
Yes	112	5.6	80	5.0	1.29 (0.94–1.77)	0.11	1.11 (0.78–1.58)	0.55
Source: self	50	2.5	22	1.4	2.03 (1.18–3.49)	0.01	1.59 (0.82–3.10)	0.17
Source: partner	31	1.6	34	2.1	0.86 (0.51–1.45)	0.58	0.92 (0.54–1.56)	0.75
Source: not known or other	31	1.6	24	1.5	1.26 (0.71, 2.24)	0.44	1.05 (0.56–1.98)	0.87
Not known if infertile	76	3.8	14	0.9	1.53 (0.80–2.93)	0.20	1.40 (0.73–2.70)	0.31
Total	1998	100.0	1597	100.0				

CI, confidence interval

^a^Adjusted for age, socio-economic status (Acorn score [20]), year of interview, marital status and geographical region of residence

^b^Excluding 11 men with Klinefelter syndrome, 9 with potentially confounding prior cancers, 29 who were severely obese at age 20 (2) or at age 40 (27), and 169 with testicular diseases

One thousand, six hundred and fifteen (80.8%) cases and 1423 (89.1%) controls had fathered any children (Table 3). There was a statistically significantly raised risk (OR = 1.50 (1.21–1.86), *p* < 0.001) for childless men. Analysis by number of children showed a highly statistically significant inverse trend if zero was included as a value (*p* < 0.001) but a borderline statistically significant trend if it was not (*p* = 0.04). These results remained statistically significant when men with Klinefelter syndrome were excluded (not in table), when in addition potentially confounding conditions were excluded (Table 3), and when analyses were restricted to married men (for childless men *p* < 0.001; not in Table). There was no relation of risk to age of the men at birth of their first child (Table 4). When infertility and number of children were fitted together (not in Table), the trend with number of children was inverse (*P* = 0.04) and the OR for men who reported that they were diagnosed as the source of infertility was less raised (OR = 1.68 (0.96–2.93)), and there were modest changes for men whose partner was the source of infertility (OR = 0.76 (0.45–1.29)) or for whom no source was identified (OR = 1.12 (0.63–2.01)).

**Table 3:** Risk of breast cancer in men in relation to number of biological children

	Cases No. %		Controls No. %		Odds ratio^a^ (95% CI) All subjects	*P*	Odds ratio^a^ (95% CI) excluding potentially confounding conditions^b^	*P*
Fatherhood (i.e. any children)								
No	383	19.2	174	10.9	1.00^c^		1.00	
Yes	1615	80.8	1423	89.1	0.67 (0.54–0.82)	< 0.001	0.70 (0.56–0.88)	0.003
No. of children								
0	383	19.2	174	10.9	1.00		1.00	
1	296	14.8	172	10.8	0.94 (0.71–1.25)	0.69	0.97 (0.73–1.31)	0.94
2	789	39.5	798	50.0	0.60 (0.48–0.75)	< 0.001	0.64 (0.50–0.81)	< 0.001
≥ 3	530	26.5	453	28.4	0.66 (0.52–0.84)	< 0.001	0.69 (0.54–0.89)	0.006
Linear trend per child, including 0					0.85 (0.79–0.92)	< 0.001	0.86 (0.80–0.94)	< 0.001
Linear trend per child, excluding 0					0.89 (0.79–1.00)	0.04	0.89 (0.79–1.00)	0.053
Total	1998	100.0	1597	100.0				

CI, confidence interval

^a^Adjusted for age, socio-economic status (Acorn score [20]), year of interview, marital status and geographical region of residence

^b^Excluding 11 men with Klinefelter syndrome, 9 with potentially confounding prior cancers, 29 who were severely obese at age 20 (*n* = 2) or at age 40 (*n* = 27), and 169 with testicular diseases

^c^ i.e. odds ratio raised (1.50 (1.21–1.86); *p* < 0.001) for childless men, if fathers are taken as the baseline

**Table 4:** Risk of breast cancer in men in relation to age at birth of first biological child

Age at birth of first child (years)	Cases No. %		Controls No. %		Odds ratio^a^ (95% CI) All subjects	*P*	Odds ratio^a^ (95% CI) excluding potentially confounding conditions^b^	*P*
No children	383	19.2	174	10.9	1.00		1.00	
< 20	65	3.3	30	1.9	1.14 (0.70–1.88)	0.60	1.33 (0.79–2.24)	0.28
20–4	426	21.3	300	18.8	0.79 (0.61–1.01)	0.06	0.82 (0.63–1.07)	0.15
25–9	588	29.4	614	38.4	0.57 (0.45–0.72)	< 0.001	0.60 (0.47–0.77)	< 0.001
30–4	317	15.9	344	21.5	0.56 (0.43–0.72)	< 0.001	0.59 (0.45–0.78)	< 0.001
≥ 35	169	8.5	117	7.3	0.84 (0.61–1.16)	0.30	0.88 (0.63–1.23)	0.47
Age not known	50	2.5	18	1.1	1.48 (0.80–2.71)	0.21	1.71 (0.88–3.30)	0.11
Linear trend per 5 years of age^c^					0.97 (0.91–1.04)	0.38	0.96 (0.89–1.03)	0.27
Total	1998	100.0	1597	100.0				

CI, confidence interval

^a^Adjusted for age, socio-economic status (Acorn score [20]), year of interview, marital status and geographical region of residence

^b^Excluding 11 men with Klinefelter syndrome, 9 with potentially confounding prior cancers, 29 who were severely obese at age 20 (2) or at age 40 (27), and 169 with testicular diseases

^c^ Excluding no children category

Sensitivity analyses excluding men whom the interviewers considered to be relatively unreliable gave similar results to those above, and in particular risks for men who were themselves the cause of the couple’s infertility (*p* = 0.04), and for men with no children (*p* < 0.001), remained statistically significantly raised (not in Table). Sensitivity analyses adjusting additionally for alcohol consumption, smoking, liver disease and family history of breast cancer (see Methods) did not change the results materially (not in Table).

In analyses separately for invasive (*n* = 1838) and in situ (*n* = 160) tumours (Table 5), the relations above were present and statistically significant for the former (unsurprisingly since they were > 90% of the total), but odds ratios were generally closer to 1.0, and in no instance statistically significant, based on much smaller numbers, for the latter. There were too few tumours that were ER-ve to conduct analyses by ER status (Table 1), but results for HER-2 + ve (*n* = 187) and − ve (*n* = 1376) tumours separately each gave similar results, with statistically significantly raised risks for male infertility (*p* = 0.04 and 0.01, respectively) and for having no children (*p* = 0.01 and < 0.001, respectively) (not in Table).

**Table 5:** Risk of invasive and in situ breast cancer in men in relation to infertility and number of children

	Invasive							In situ				
	Cases No. %		Controls No. %		Odds ratio^a^	(95% CI)	*P*	Cases No. %	Controls No. %	Odds ratio^a^	(95% CI)	*P*
Infertility												
No	1664	90.5	1503	94.1	1.00			146	1503	1.00		
Yes	107	5.8	80	5.0	1.34	(0.97–1.85)	0.07	5	80	0.70	(0.28–1.79)	0.46
Not known	67	3.6	14	0.9	1.55	(0.79–3.02)	0.20	9	14	2.31	(0.88–6.10)	0.09
Source												
Self	47	2.6	22	1.4	1.96	(1.13–3.41)	0.02	3	22	1.72	(0.50–5.94)	0.39
Partner	30	1.6	34	2.1	0.93	(0.55–1.58)	0.81	1	34	0.28	(0.04–2.09)	0.21
Not known	30	1.6	24	1.5	1.35	(0.75–2.44)	0.31	1	24	0.53	(0.07–3.98)	0.53
No. of children												
0	353	19.2	174	10.9	1.00			30	174	1.00		
≥ 1	1485	80.8	1423	89.1	0.65	(0.52, 0.81)	< 0.001	130	1423	0.87	(0.54, 1.41)	0.58
1	267	14.5	172	10.8	0.93	(0.69–1.24)	0.60	29	172	1.38	(0.76–2.52)	0.29
2	733	39.9	798	50.0	0.60	(0.48–0.76)	< 0.001	56	798	0.66	(0.39–1.12)	0.12
≥ 3	485	26.4	453	28.4	0.63	(0.49–0.81)	< 0.001	45	453	0.99	(0.57–1.71)	0.97
Linear trend per child, including 0					0.84	(0.78–0.91)	< 0.001			0.93	(0.78–1.12)	0.44
Linear trend per child, excluding 0					0.88	(0.78–0.99)	0.04			0.95	(0.71–1.26)	0.71
Total	1838	100.0	1597	100.0				160	1597			

CI, confidence interval

^a^Adjusted for age, socio-economic status (Acorn score [20]), year of interview, marital status and geographical region of residence

## Discussion

Our large case–control study has shown a clear, statistically significant, association between reported diagnosis of male infertility and risk of breast cancer, and this was supported by analysis of numbers of offspring—there were significantly more men with no children among cases than among controls, both overall and after excluding potentially confounding conditions, and in analyses restricted to married men.

Case–control studies are notoriously at risk of bias, but there seems no plausible reason why our infertility results should have been artefactual. Although our controls, unlike the cases, were not strictly population-based, because of the poor response rate now for population-based controls [16, 17], they were drawn from the whole country and age range of the cases, we adjusted for the difference in distribution of relevant variables between the cases and controls, and the infertility results were present in comparisons with each of the two control groups, from different sources, used in the study. The association with infertility is not one plausibly known to the subjects, and there is no obvious reason why the men should have recalled or reported it in a way biased between cases and controls, especially for reporting of number of children. We could not interview patients who died before they could be approached or interviewed, which could lead to bias if survival was related to infertility, but there is no obvious reason why it should be nor any evidence for such an association to our knowledge. Although a small part of the association is explicable by the known association of male breast cancer with Klinefelter syndrome [21], this is far too rare to account for the overall relation, which persisted after excluding subjects with known Klinefelter, based on cytogenetic karyotyping of the first 901 cases and self-reporting of diagnosis for the remainder. The association also largely remained after additional exclusion of patients with other pre-existing potential confounders, namely severe obesity, past malignancies that can be treated with chest radiotherapy, and testicular abnormalities [22]. It is arguable, however, whether the latter exclusions are overly conservative, since several of the testicular conditions, which were the main exclusions, may have been diagnosed as a consequence of investigations for infertility rather than diagnosed independently of it: we did not have information to determine the sequence of diagnostic dates. There are several other factors that are known or posited to be related to risk of infertility, including diabetes, dyslipidaemia, cirrhosis of the liver, alcohol consumption, smoking, and endocrine disrupting chemicals [7], but there is no convincing evidence that these factors are related to risk of breast cancer in men [2, 8–15, 23], and hence, no reason to believe that they are confounders for which adjustment would be needed in our infertility analyses. Conversely, known risk factors for breast cancer in men, family history of breast cancer and risk genotypes [1] are not known to be associated with risk of infertility and hence again are not clear confounders. Nevertheless, in sensitivity analyses, adjustment for those of these variables for which we had data made no material difference to the results.

Self-reported fertility is a ‘soft’ measure with potential for misclassification for several reasons. Infertility is a complex process that can include factors from both the male and female members of a couple; men may not report (or even know of) children born outside marriage; they may have remained childless by choice, not infertility; and they may report a low sperm count even if it was not the reason for infertility. Additionally, the self-reports were reliant on the men’s recall and understanding—the evidence would have been stronger if infertility could have been validated from medical records, but this was not practical across an entire country over many decades. However, although all of the above sources of misclassification might plausibly have led to dilution and hence underestimation of any true risks, there is, as noted above, no obvious reason why this should have been differential between cases and controls, and hence have led to bias.

The lack of a significant association of male breast cancer with infertility in most of the previous literature does not argue substantially against the association that we found: the few published studies [8–10] have had mixed results, with at most only 227 cases of whom 7 reported infertility [8]. A pooled analysis that included 420 cases [2] found a non-significant odds ratio of 1.36, although based on heterogeneous case–control studies with heterogeneous definitions of infertility that complicate interpretation, and with analysis solely of a dichotomy between “history of infertility” and no such history.

Our finding of greater risk for men with no biological children compared with any children is congruent with infertility as a risk factor. Most previous studies have not found such an effect, but based on very small numbers [11, 12, 15, 24], although one small study [13] (21 cases) and a pooled analysis [2] found significantly raised risk for men with no children.

We found significantly decreasing risk with increasing numbers of children. However number of children beyond one is difficult to interpret as an indicator of male fertility, since it may more reflect social and cultural factors than fertility per se. The same is true for age at first birth. Similarly, it is difficult to interpret analyses confined to married men because of the changing relation of marital status to fatherhood in Britain, such that the meaning of ‘married’ in relation to potential for fatherhood has changed over time. Before 1980, 10% or fewer of births in England and Wales were outside marriage, but the proportion has since soared such that by 2000, 40% were outside marriage and subsequently almost 50% [25]. In our main analyses, we adjusted for marital status; in comparison, analyses confined to married men showed slightly less marked odds ratios, but entirely in the same direction.

Our data showed that the association of infertility with male breast cancer risk was clearly present for invasive tumours, but not significant, based on much smaller numbers, for in situ tumours: this does not appear to have been investigated previously.

The reason for the association of male infertility with breast cancer risk demonstrated in our data is uncertain. Infertility can result from a wide range of factors, including genetic, congenital anomalies of the genitourinary tract, other anatomical reasons, and sexual dysfunction, but most cases are idiopathic [22].

The main source of testosterone secretion in men is the testis, so one potential link between infertility and breast cancer risk would be via hormonal effects of testicular abnormalities. For instance, mumps orchitis can lead to testicular atrophy and long-term reduced testosterone production [26], and can lead, albeit not commonly, to subfertility or, rarely, sterility [27]. Although significant associations remained (albeit slightly reduced) after exclusion of men diagnosed with testicular abnormalities that have been reported to be associated with breast cancer risk, it is possible that associations remained from other abnormalities of the testes not know to be risk factors, or more subtle abnormalities that would not receive a diagnosis.

It has been hypothesised that prenatal oestrogen exposure can lead to raised risk of male infertility [28], in which case a possible connection would be if the prenatal hormone environment might also affect male breast cancer risk, as hypothesised for women [29].

## Conclusions

In summary, our large case–control study gives strong evidence that male infertility is associated with raised risk of breast cancer in men. The reasons are uncertain and need to be investigated.

## Data Availability

The statistical output data underlying this article will be shared on reasonable request to the corresponding author. The individual subject data underlying this article cannot be shared because of the privacy of the individuals who participated in the study.

## Abbreviations

OR: Odds ratio
CI: Confidence interval
ER: Oestrogen receptor
HER2: Human epidermal growth factor receptor 2
